# *To live in the present* – adapting to life with advanced heart failure: a qualitative interview study

**DOI:** 10.1080/17482631.2025.2571330

**Published:** 2025-10-30

**Authors:** Elisabeth Gustavsson, Marie-Louise Södersved Källestedt, Åsa Revenäs

**Affiliations:** a Section of Cardiology, Department of Medicine, Region Västmanland, Västerås, Sweden; b Centre for Innovation, Research and Education, Region Västmanland, Västerås, Sweden; c School of Health, Care and Social Welfare, Mälardalen University, Västerås, Sweden; d Orthopedic Clinic, Region Västmanland, Västerås, Sweden

**Keywords:** Heart failure, palliative care, end-of-life care, quality of life, communication, nursing

## Abstract

**Background:**

Despite evidence that structured, multidisciplinary care for people with advanced heart failure (AHF) significantly enhances patients’ quality of life, critical questions remain regarding how such care should be optimally designed. To improve the quality of life for people with AHF, healthcare professionals must deepen their understanding of what these patients genuinely need and how best to meet them in their illness trajectory.

**Aim:**

To describe patients’ experience of living with advanced heart failure in Sweden.

**Methods:**

Fifteen patients with AHF from a medium-sized hospital in Sweden were recruited for a qualitative interview study. Content analyses were conducted.

**Results:**

Living with AHF was viewed as life changing. The overarching theme, *To live in the present,* describes the participants’ overall experience of their life situation. Four categories and ten subcategories were identified. The four categories were: *Symptoms affecting life, Making the most out of life given, Coping with emotions,* and *To be a part of health care*.

**Conclusion:**

This study provides valuable insights in how patients with AHF experience their life and need care. The results highlight the need for health care to provide support to help patients cope with emotional, social, spiritual and practical everyday life situations to ease self-care.

## Introduction

Circulatory diseases, such as heart failure (HF), are the leading cause of death in Europe. The prognosis is severe but has improved in recent years. Despite this improvement, 53−67% of people with advanced heart failure (AHF) are at risk of dying within five years, and only a minority receive palliative care (McDonagh et al., 2021).

HF occurs in two percent of the population in Sweden (as in Europe and USA) and for people over 80 years the prevalence is 10%. HF increases the risk of a premature death and reduces quality of life (Jaarsma et al., 2009). According to the New York Heart Association (NYHA), AHF (NYHA class III–IV) can be described as the persistence of severe signs and symptoms of HF, despite optimal evidence-based treatment (The Criteria Committee of the New York Heart Association. Nomenclature and Criteria for Diagnosis of Diseases of the Heart and Great Vessels, 1994). Physical symptoms of heart failure are numerous (McDonagh et al., 2021), with the most common ones being shortness of breath, cough and swelling (Ferrell et al., 2015; Katz & Konstam, 2009). Fatigue (Adler et al., 2009; Ferrell et al., 2015), insomnia (Whellan et al., 2014), loss of appetite (Ferrell et al., 2015) and pain (Evangelista et al., 2009) are also common. The characteristics of HF are that the patient has long periods without symptoms and then enters a period of cardiac deterioration. The course of the disease is unique to each patient and includes recurrent unpredictable deterioration (Jaarsma et al., 2009). HF patients report psychological symptoms, psychosocial burdens, and spiritual needs that are consistent with reports of people with other chronic diseases, such as advanced cancer (Sobanski et al., 2020). Studies have shown that 21–36% of HF patients have depression, and in patients with advanced HF, the incidence of depression is even greater (Adler et al., 2009).

Guidelines recommend that multidisciplinary management programs are necessary to achieve optimal management of HF (McDonagh et al., 2021). Nurse-led HF clinics have existed in Sweden since the 1990s and today, most hospitals in Europe and the US have their own HF clinic (Qui et al., 2021; Strömberg et al., 2001). Nurses at these clinics titrate HF drugs, conduct structured follow-ups and provide education for patients. The goal of HF clinics is to improve patients’ quality of life, drug compliance and self-care and to reduce morbidity and hospitalisation (Strömberg, 1998).

The last six months of the HF patient’s life are often characterized by frequent hospital admissions, culminating in an in-hospital death (Unroe et al., 2011). Each HF-related hospitalization increases the risk of the next event. Worsening congestion and signs of fluid overload remains the major cause of hospitalisation (Tomasoni et al., 2011).

Early palliative care is recommended for people with AHF to improve their quality of life and ability to die, according to the European Society of Cardiology (ESC) (McDonagh et al., 2021). Across available studies consistently less than ten percent of patients with HF ever receive palliative care services (Brännström et al., 2012; Gelfman et al., 2024; Liu et al., 2020). Palliative care focus on an individual’s needs and personal choices. The care should strive to offer painlessness, dignity and peace (Sobanski et al., 2020). Guidelines recommend that healthcare professionals engage in an open dialogue with patients and their relatives about the course of HF, including their wishes for future care and awareness of the risk of sudden death (McDonagh et al., 2021). Similarly, it is important that healthcare professionals adapt their communication to patients’ needs (Hjelmfors et al., 2018). It is highly important for HF patients to be open to meeting between patient and staff to achieve a sense of security (Söderberg et al., 2015).

Despite the evidence that structured, multidisciplinary care for HF significantly enhances patients’ quality of life (Jaarsma et al., 2009; Schichtel et al., 2019), critical questions remain regarding how such care should be optimally designed for individuals with AHF. The experiences and perspectives of these patients are still insufficiently explored in the literature. To provide truly person-centred and meaningful care, healthcare professionals must deepen their understanding of what these patients genuinely need and how best to meet them where they are in their illness trajectory.

### Aim

The aim of this study was to describe patients experience of living with advanced heart failure in Sweden.

## Methods

### Study design and ethics

This interview study had a qualitative descriptive design. The interviews were conducted between September 2021 and June 2022 and analysed following a content analysis as described by Lundman and Hällgren Graneheim (2017). A semi-structured interview guide was developed by the authors based on their experience and the study aim. Ethical approval was provided by the Swedish ethical review authority in Uppsala (Dnr 2021-03589). The participants provided both oral and written informed consent.

### Participants and recruitment

Fifteen patients from a medium-sized hospital in Sweden with both urban and rural areas and a catchment area of ​​approximately 180,000 people participated in the study. Nurses (none of whom were the authors of the study) from the HF clinic identified potential participants among their patients with NYHA class III–IV and no other life-threatening illness. The participants must speak and understand Swedish and be willing to be interviewed in an audio-recorded interview. Patients with cognitive impairment, such as having memory problems, were excluded.

A purposive sampling was used, and a variation in age and time with HF diagnosis was sought. The HF nurses informed 19 patients about the study. Those who agreed to participate approved their name and phone number being shared with the researcher. The researcher called the potential participants to inform them about the study and book an interview. During the phone call, the participants gave verbal consent.

### Data collection

The interview guide was constructed by the research team (EG, MLSK and ÅR) and guided by the aim of the study. The guide was pilot tested by the EG in the first interview with a patient with AHF. After discussion, the researchers concluded that the interview guide was sufficient to answer the research aim. No changes were needed. The interview guide consisted of questions about the patients’ experiences with everyday life and care living with AHF (see Interview guide, Supplementary file 1). Individual face-to-face interviews were performed (duration between 6 and 23 min) and audio recorded. The interviews were held in the HF clinic in the room where the patients usually visited the HF nurse. All the participants were given verbal information about the study and provided written consent before starting the interviews. Audio recordings and transcribed interviews were securely stored digitally in a password protected location and in accordance with the research principal's guidelines. All the interviews were conducted by the first author (EG), who is a registered nurse with more than 5 years of experience treating patients with HF. Data collection was decided by the researchers to close after the 15th interview when no new information was disclosed.

The authors have previous experience in qualitative research as well as clinical experience in cardiac, intensive and orthopaedic care, through which all patients with HF pass. Given our diverse professional backgrounds, two nurses and one physiotherapist, we are aware that our perspectives could influence both data collection and interpretation. None of the authors had any dependency or prior relationship with the interviewed patients. During data processing and analysis, we applied continuous reflexivity, including ongoing self-reflection, peer discussions, and critical dialogue. This reflexive approach helped us remain aware of our positionality and potential biases, thereby contributing to a transparent and trustworthy analytical process. The interdisciplinary collaboration enabled us to focus on the research question and approach the data from complementary perspectives.

### Data analysis

The audio-recorded interviews were transcribed by the first author. The text was analysed using qualitative content analysis as described by Lundman and Hällgren Graneheim (2017). The interviews were read multiple times to identify domains. The meaning units with meaning-bearing parts of the text were lifted out of the content and context of the text and then condensed. The condensed meaning units were coded through abstraction, and codes with similar content were grouped into categories. The analysis was performed manually, using post-it notes for individual codes and categories. Later, in the analysis process, the codes and categories were summarized in a word table. To promote trustworthiness, categories and subcategories were identified, discussed, and refined several times until the authors reached consensus (Lundman & Hällgren Graneheim, 2017). The discussion theme was derived, which interlinked the categories and described the participants’ overall experience of their life situation and care.

## Results

Of the 19 participants who were informed about the study, 15 consented to participate. Patient demographic details are presented in Table I. None of the participants were candidates for heart transplantation or left ventricular assist devices (LVADs).

**Table I.** Demographic characteristics of the participants.

Gender	*N* (%)
Male	10 (67%)
Female	5 (33%)
Age	Year
Median	77
Range	53−89 years
Marital status	*N* (%)
Married or living together with someone	12 (80%)
Living alone	3 (20%)
Time with diagnosis	0−11 years
Median	2 years
NYHA classification	NYHA III: 15 (100%)
No resuscitation decision	3 (20%)

The overarching theme, *To live in the present*, describes participants’ overall experience of living with AHF. Four categories and ten subcategories were identified. The categories were: *symptoms affecting life,* and the symptoms of AHF that had the greatest impact on participants’ lives were lack of physical energy, fatigue and fluid overload. *Making the most out of life given,* AHF had left them feeling limited in their everyday life but also made them adjust their lives to try to do the best with what they have. *Coping with emotions*, fear and loneliness were feelings that the participants had to manage when trying to cope with their life. *To be a part of health care,* participants described that they received care based on their needs and felt safe. How information about prognosis and discussions about goal-oriented care was communicated was considered important (Table II). Quotations are included to further illustrate participants’ experiences.

**Table II.** Categories and subcategories presenting participants’ experiences of their life situation.

The theme: To live in the present	
Categories	Subcategories
Symptoms affecting life	Less energy
	Shortness of breath and swelling
Making the most out of life given	Adjusting to life
	Hoping for improvement
Coping with emotions	Fear of not being able to manage
	Feeling of loneliness
To be a part of health care	To obtain honest information
	To involve relatives
	Care based on needs

### Symptoms affecting life

The symptoms of HF that had the greatest impact on participants’ lives were lack of physical energy and fluid overload. The degree of impact varied among the participants; some described that they felt that they had improved, while others felt that their condition had worsened.

*Lack of energy*

The participants mentioned a lack of energy as a struggle. They declared that they had no remaining physical strength, a feeling of major loss of energy and exhaustion. The participants described that they used to be active and do certain chores, but now the chores were impossible. They could not even cope with the daily chores at home. Making the bed could be an adventure and tying the shoes a ‘gym session’. If they could do something, the action went much slower than before.

’*You think you can handle it, so I’ve been out driving the lawnmower a bit, but then I’m completely exhausted. So, then a whole day goes by when you can’t do anything more.’* (P15)

At the same time, the participants described a fear of being inactive. They understood the importance of being active and forced themselves to exercise.

*‘I forced myself to walk because I thought it was something with the muscles. That you must walk, otherwise the muscles wither*.’ (P10)

The participants mentioned the importance of being active to feel that they were doing something to improve their lack of physical energy.

A major loss of energy and exhaustion was expressed among the participants. Things they thought they would be able to do in life turned out to be impossible because of the HF.

*‘I have been very energetic in my life but now I can’t take it anymore.’ (P1)*

They used to engage in projects and with people but now they described not being able to become involved in the same way.

*Shortness of breath and swelling*

The participants also described symptoms of fluid overload, such as shortness of breath and swelling, as a significant problem.

‘*I get short of breath quite often, as soon as I do something … As soon as I have to increase the pace or walk uphill, I feel it at once.’* (P7)

Fluid overload was mentioned as a symptom that made participants seek medical care.

‘*There is a lot of fluid accumulating in my body, so I have to go up* (to the hospital) *to have it drained at regular basis.’* (P9)

### Making the most of life given

The participants felt despondent and thought that having AHF was the worst thing they had ever experienced. AHF had left them feeling limited in their everyday life but also made them adjust their lives to try to do the best with what they have.

*Adjusting life*

When the participants discussed their life with AHF, they mentioned that they needed to adjust their life. Most of them felt limited and had to sift through what they could and could not do. The participants explained that they were afraid that something would happen and that they hardly dared to leave home. They also had to ask for help with things they were previously able to do themselves.

*‘I can’t really do what I want, I have to get help with things I’ve always done myself before.’* (P7)

Adjusting to the new life was described as difficult but viewed as fundamental. They said that they need to prioritize when they cannot do everything they want to do. They also described that they did not recognize themselves and that they felt ashamed for not being as active as before. The participants had to involuntarily change their way of living.

*Hoping for improvement*

Nevertheless, while the participants expressed feelings of being in bad shape, they hoped for improvement and were willing to make the most of their life.

*‘I think that you live for such a short time, but you are dead for a damn long time. It is important to take advantage of that moment.’* (P3)

The participants described that if they just could be a little bit better, they could live with HF other participants realized that they would never have improved but had to live with it and cope in the best possible way. The participants also described how they did not want to look ahead. Participant 9 expressed the following: *‘The future is tomorrow’*.

In the absence of becoming better, participants took one day at a time.

### Coping with emotions

Participants discussed going through crises, and newly diagnosed participants felt like they were in shock. Fear and loneliness were feelings the participants had to manage when thinking about the last time in life.

*Fear of not being able to manage*

The participants expressed fear of being bedridden and unable to take care of themselves. Participant 1 was fully aware of not becoming any fitter.

*‘It is just a matter of keeping it* (not being able to manage himself) *away as long as possible to avoid being bedridden’.*

Participants thought a great deal about how the last time in life would be if they could not take care of themselves. They were also afraid of having to suffer in their last time in life.

*Feeling of loneliness*

Participants expressed that they felt insecure due to relatives’ ignorance. Some thought it was difficult to discuss the disease and prognosis with relatives and friends and therefore felt isolated. Others did not want to worry their relatives and therefore did not tell the relatives how they felt. Those who lived alone expressed that they felt vulnerable when they were at home.

*‘In the beginning I simply didn’t dare to be alone.’* (P7)

The feeling of loneliness was described as present in the participants’ lives regardless of whether they lived with someone or alone. When they had no one at home they could talk to, the participants turned to the internet and Facebook group, in which they found people in similar situations.

### To be a part of the health care

The participants described that they received care based on their needs and felt safe with regular check-ups, such as an inspection. How information about prognosis and discussions about goal-oriented care were communicated was considered important.

*To obtain honest information*

The participants expressed a desire to know about their illness and prognosis. Honesty and clarity were something they demanded.

*‘It doesn’t matter if it’s a bad prognosis or a good one, just so I know.’* (P9)

No one wanted information withheld, but consideration was requested when information was presented. Importantly, the information was repeated, as many participants could not assimilate all the information the first time. In discussions about goal-oriented care, some participants thought about death, and the conversation was natural. They viewed the discussions as an opportunity to discuss how they wanted their last period of life.

*‘I have talked to my doctor, if this* (heart attack) *happens again, I just want life to stop.’* (P14)

Others sensed that death was approaching, but no one had spoken to them about this. Others felt that they could not bear to think about the future and expressed that they were not ready or interested to discuss it at the time.

*To involve relatives*

Participants mentioned that bringing a relative or friend to the discussion about prognosis and goal-oriented care was a good idea to help assimilate the information provided. The participants thought it was important to involve relatives in their care. They mainly mentioned partners and children when they talked about relatives, but someone talked about a close friend.

*‘You mustn’t forget to involve the next of kind … it’s super important.’* (P5)

Participants stated that relatives who received information and felt involved with the care could help and support them better.

*Care based on needs*

Participants were grateful for the treatment they received from the healthcare organisation and expressed that they felt a great sense of security and trust in the staff. When participants came to the HF clinic on a regular basis, they felt calm and safe. They believed that with the nurses, they could raise matters that were not related to their care or their illness, small things that nevertheless felt significant to them. They also always felt comfortable asking questions and requesting information.

*‘I am lucky to have her (HF nurse), I have been able to open up. Talk about things that maybe she shouldn’t sit and listen to, but it has been a security that she has been there.’* (P11)

Participants often called themselves “regulars” at the HF clinic. The knowledge of being able to call the clinic every day and come to see a nurse or doctor at short notice provided security.

Sometimes, participants felt uncertain, especially when there was poor continuity.

‘*It feels like I’m jumping to a new doctor and then it’s a new one and then I’m going to see a new doctor again today …. I felt safe when I came to the heart failure nurse. It feels like the nurses have been very capable, the doctors have rushed by.’* (P2)

The participants described that the HF clinics are accessible to them and, in that way, contribute to security.

## Discussion

Participants in this study described how they struggle with their unpredictable life with AHF and how living in the present was one way to cope with the situation. They faced different challenges that were both unique and similar. They felt despondent and limited but also tried to adjust and make the most of life given. The greatest fear was to be unable to take care of themselves and to suffer during their last life. To obtain honest information and involve relatives in care was described as an important part of dealing with life with AHF.

Participants described that living with AHF had made them feel that they were not able to recognize themselves. They realized that they were no longer the same person and were unable to perform daily tasks or were forced to give up what they enjoyed before. This type of identity crisis has been observed in several other studies when people have been diagnosed with a chronic disease (Cheryl et al., 2021; Nordfonn et al., 2019), and many struggle to maintain their self-identity (Cheryl et al., 2021). The change from being a healthy person to being a severely ill person has been described previously as emotionally difficult and has been called a “before-and-after experience” (Nordfonn et al., 2019). Despite the limitations, participants in our study experienced the need to accept the situation and live in the present. To take one day at the time and not to plan too far ahead and to appreciate and make the most of the “good” days seems to be one coping strategy to manage the identity crisis.

The possibility of easily contacting the HF clinic and regular visits was described to be of the utmost importance to feel safe. This observation is in line with previous studies that described how regular visits, telephone calls and the continuity of personnel provided a sense of security (Kitchens et al., 2015; Talabani et al., 2020). A meta-analysis from 2020 showed that nurse-led follow-ups had a significant impact on reducing the risk of rehospitalization and mortality in patients with HF (Kitchens et al., 2015). The feeling of being taken care of by skilled personnel provided the participants with hope in another study (Schaufel et al., 2011). The fact that the participants had the opportunity to call or see a HF nurse or doctor on short notice made them feel safe and speculatively, it could lead to fewer visits to emergency care.

As in previous research (Barclay et al., 2011), the participants in this study emphasized that they wanted information sensitively, with honesty and repeated opportunities to talk. Ineffective communication on prognosis and patients’ needs increases only patients’ anxiety and uncertainty and affects clinical outcomes (Stömberg & Jaarsma, 2008). A greater focus on communication and early patient involvement in the decision-making process has been shown to be important for patients’ self-management and their health outcomes (Rognan et al., 2023). Patients want to discuss their prognosis and express a desire to be informed and participate in their own healthcare decision (Garland et al., 2013). The participants in our study had, on the other hand, different views on discussions about death and their last period of life. The participants who did not want to discuss this topic were all younger and newly diagnosed. The participants in our study did not express any death anxiety but were worried about not being able to cope and suffer in the final stages of life. This finding is in line with previous research, which revealed that only 16% of HF patients are afraid of dying and that they are more afraid of deterioration and a painful and difficult time before death (Stömberg & Jaarsma, 2008). The patients live from one week to another, not looking too far ahead (Schaufel et al., 2011). Many patients prefer better quality of life over prolonged quality of life (Ischanow et al., 2022). Furthermore, previous studies have shown that it is important to start addressing existential and goal-oriented care issues before HF becomes too advanced (Stömberg & Jaarsma, 2008). To address this stigma and begin discussing prognosis and palliative care early in the disease course, the term “discussion in case of severe illness” was introduced in Swedish palliative care (Nationellt system för kunskapsstyrning Hälso- och sjukvård, 2022). This term makes recurrent discussions about palliative care possible during the unpredictable course of the disease. However, communication about palliative care is shaped not only by medical uncertainty but also by personal and cultural contexts. A developed and inclusive language, ideally supported by visual aids, is crucial to ensure that patients from diverse backgrounds can engage meaningfully in conversations about heart failure (Alassoud, 2022). Further studies are needed to deepen our understanding of these sociocultural dimensions.

Our participants felt lonely and insecure and found it difficult to talk to family and friends. They stated that it is important to involve relatives in care to ease their participation and support in care. Similarly, a previous study showed that HF patients feel like a burden on their family because of the disease and the associated new demands of self-care (Strömberg, 1998). Moreover, life at home is associated with feelings of loneliness in being severely ill even when patients are surrounded by people (Strömberg, 1998). One way to minimize feelings of loneliness may be to talk to others in similar situations, as mentioned by participants. A previous study on peer interaction demonstrated that contact with others with HF is a source of emotional support that provides the opportunity to talk with, share and learn from other people with HF. Interaction with other people living with HF has been described as helping patients realize that they are not alone (Lockhart et al., 2014). Participants in our study had no opportunity to participate in group gatherings. Instead, they turned to Facebook groups and other contexts on the Internet. To encourage and facilitate patients to talk to peers should be considered an option in the care of patients with AHF.

Further studies should investigate the role of peer interaction and whether it may reduce loneliness and support emotional well-being in heart failure care.

Limitations and strengths

This study involved only a small number of participants. However, in qualitative studies, credibility has more to do with the richness of information in each interview (Lincoln & Guba, 1985). To ensure the credibility of the analysis, all the authors read and discussed the content several times during the analysis process. The study does not include quantification or claims of causality since this is not the intention with a qualitative design. Rather, its purpose is to describe in depth the lived experience of patients with AHF. Unfortunately, there were no participants with NYHA class IV disease in this study. The reason may be that they are fewer in number in a heart failure clinic, as they receive more help at home or are admitted to a ward. Since these patients are sicker in their HF, their experience would have been valuable in this study.

Moreover, the interviews were generally quite short, one only 6 minutes long. The reason for this may have been the difficult and sensitive topic. Even though the interviews were short, we believe the content is rich enought to contribute to the understanding of the lived experience of AHF. Hence, the results of this study may be transferable to patients with NYHA classes III and IV with a symptom burden similar to that of the participants in this study.

More studies in different locations are needed to reveal the extent to which the results are transferable to patients admitted to other HF clinics, which may have different care organizations.

This study has several strengths. First, we found that the participants were representative of the population of patients with AHF. Second, all interviews followed the same interview guide and were performed by the same researcher, ensuring that the same topics were handled in the same way. Theme and categories were discussed several times throughout the analysis process by all the authors to ensure trustworthiness. Quotations from the interviews are presented in the results, offering the reader the opportunity to judge the trustworthiness of the findings.

Finally, the authors have previous experience in qualitative research as well as clinical experience in cardiac, intensive and orthopaedic care, through which all patients with HF pass.

## Conclusion

This study describes participants’ experience of living with AHF. The theme of living in the present describes the participants’ overall experience of their life situation. The participants described living with uncertainty and identity loss and described feelings of isolation and fear of losing independence. Coping strategies included living in the present and valuing good days. Honest communication and goal-oriented care discussions, the involvement of relatives, and easy access to healthcare professionals provided a sense of safety.

Our study suggests that it is important for AHF patients to have easy access to healthcare professionals to feel secure and potentially reduce the need for emergency visits or acute admissions.

The results highlight the need for health care to provide support to help patients cope with emotional, social, spiritual and practical everyday life situations. However further studies are to investigate the role and potential of relatives and peer support and if that may reduce loneliness and support emotional well-being in advanced heart failure care.

## Supplementary Material

Supplementary material Supplementary file 1_nterview guide_rev1

## Data Availability

Pseudonymized data may be available from the corresponding author upon request and subject to GDPR and Swedish Ethical Review Authority requirements.
