# Supplementary material for: To live in the present – adapting to life with advanced heart failure: a qualitative interview study
Source: Int J Qual Stud Health Well-being. 2025 Oct 30;20(1):2571330. doi: 10.1080/17482631.2025.2571330 (PMC12581781; doi:10.1080/17482631.2025.2571330)
Supplement: Supplementary material — Supplementary file 1_nterview guide_rev1 [file ZQHW_A_2571330_SM7634.docx]

Interview guide translated from Swedish to English

The interviewer starts by thanking the participant for participating in the research study that aims to shed light on life with advanced heart failure.

The interviewer informs the participant that the interview will be audio-recorded and that individual answers from the participants will not be able to be recognized. The participation is voluntary and the interview can be discontinued at any time, without giving any reason. The participant is encouraged to ask questions about the research and then give written informed consent.

1. Opening question:

• Can you start by telling me how you are feeling right now?

The interviewer uses prompts such “tell me more”, “Can you elaborate more on…”, “Can you give example on….”

1. Question areas to cover:

• Which heart failure symptoms affect your life as a heart failure patient?

Example questions:

What symptoms do you have from your heart failure?

(Pain? Cough? Dyspnea? Swelling? Difficulty eating? Fatigue? Confusion? Difficulty sleeping? Anxiety?) 2. Does it prevent you from doing things? In what way do the symptoms affect you?

3. How do you view the future course of the disease?

Care? Care at home/hospital

How do you perceive the information about prognosis and end-of-life care that you have received from the care team as a heart failure patient?

Example questions:

4. Which possible different professional groups (doctors, nurses) have you spoken to about the heart failure prognosis and what the future may be like?

a. What was that discussion like?

b. Who/which professional groups did you talk to?

c. What did you want to talk about and what did the healthcare professionals talk about?

d. What did this conversation mean to you? What reactions did you have and what do you think about it?

e. What follow-up questions did you have? How did you ask these questions during the conversation? If you did not ask any questions, what did you not ask any questions?

Example questions:

5. There is a conversation called the Breakpoint Conversation (when treatment becomes palliative, i.e. when you only relieve symptoms), are you familiar with it? Can you briefly describe what that conversation might be about?

a. When do you feel that you would like someone to have such a conversation with you? At the beginning/when it is time.

b. What do you feel that healthcare professionals have done to make it easier for you and your situation?

c. How do you, as a heart failure patient, want care to be provided now and in the future?

d. Could anything be done differently?

6. How are your life today, please describe??

a. When do you feel secure?

b. What makes you feel secure when it comes to your illness?

c. What makes you feel insecure?

d. What feels good / not good?

7. Is there anything that we haven't talked about that you want to share with me?

8. Could you briefly summarize what you think has been most important to you during this interview.
